# Effector Mechanisms of CD8+ HLA-DR+ T Cells in Breast Cancer Patients Who Respond to Neoadjuvant Chemotherapy

**DOI:** 10.3390/cancers13246167

**Published:** 2021-12-07

**Authors:** Rubén Osuna-Gómez, Cristina Arqueros, Carla Galano, Maria Mulet, Carlos Zamora, Agustí Barnadas, Silvia Vidal

**Affiliations:** 1 Inflammatory Diseases, Institut de Recerca de l’Hospital de la Santa Creu i Sant Pau, Biomedical Research Institute Sant Pau (IIB Sant Pau), 08041 Barcelona, Spain; rosuna@santpau.cat (R.O.-G.); cgalano@santpau.cat (C.G.); mmulet@santpau.cat (M.M.); czamora@santpau.cat (C.Z.); 2 Department of Medical Oncology, Hospital de la Santa Creu i Sant Pau, 08041 Barcelona, Spain; carqueros@santpau.cat (C.A.); abarnadasm@santpau.cat (A.B.); 3 Centro de Investigación Biomedica en Red Cancer (CIBERONC), 28029 Madrid, Spain; 4 School of Medicine, Universitat Autònoma Barcelona, 08193 Bellaterra, Spain

**Keywords:** breast cancer, CD8+ T cell, cytotoxicity

## Abstract

**Simple Summary:**

This study contributes to the characterization of CD8+ HLA-DR+ T cell mediated-mechanisms involved in the anti-tumor response in breast cancer patients who respond to neoadyuvant chemotherapy (NACT). Cultures with plasma from responder (R), but not from non-responder (NR), patients increased the intracellular production of cytotoxic molecules and pro-inflammatory cytokines in CD8+HLA-DR+ T cells. However, the addition of neutralizing anti-IFN-γ or anti-IL-12 antibodies to cultures with plasma from R patients decreased this effector function on CD8+HLA-DR+ T cells. These results suggest the presence of soluble factors in the plasma of R patients inducing the effector function of CD8+HLA-DR+ T cells.

**Abstract:**

Cytotoxic T lymphocyte (CTLs) activation is an independent predictor of response to neoadjuvant chemotherapy (NACT) in breast cancer (BC) patients. Here, we go deeper into the function of CD8+ HLA-DR+ T cells from NACT treated HER2 negative BC patients. Flow cytometry analysis revealed that CD8+ HLA-DR+ T cell percentage was increased in NACT responder (R) compared to non-responder (NR) patients. R patients with ER-/PR- hormone receptors had the highest CD8+ HLA-DR+ T cell frequencies, while no differences were found when patients were classified according to cancer stage or menopause status. Interestingly, the cytotoxicity and production of anti-tumor cytokines were enhanced when CD8+ HLA-DR+ T cells from healthy donors were cultured with plasma from R, but not from NR patients. The induced anti-tumor profile of CD8+ HLA-DR+ T cells was associated with plasmatic IL-12 and IFN-γ levels, increased cytokines in R patients. IL-12 or IFN-γ neutralization decreased cytotoxic activity and TNF-α production by cultured CD8+ HLA-DR+ T cells in R plasma presence. All these data suggest that an effective response to NACT in BC patients is associated with increased IL-12 or IFN-γ levels involved in the induction of cytotoxic and pro-inflammatory mechanisms in CD8+ HLA-DR+ T cells.

## 1. Introduction

Breast cancer (BC) remains the most common malignancy and a major cause of cancer-related death in women worldwide [1,2]. Due to significant progress in early diagnosis and individualized cancer management, the clinical outcome of BC has improved in the last recent years [3,4]. Neoadjuvant chemotherapy (NACT) is recommended for patients with locally advanced BC, patients with lymph node metastasis and those who are not suitable for breast-conserving surgery [5]. Some patients achieve a complete pathological response (pCR), which has a strong indicator of disease-free survival [6,7]. In contrast, residual disease after NACT and HER2 expression are predictors of relapse [8]. Other factors that are associated with the response to NACT are: infiltration of tumor infiltrate lymphocytes (TILs), tumor size, histological type, Ki-67 and the expression of hormone-receptors [8,9].

Previous studies in triple negative breast cancer (TNBC) have shown an increase of TILs in 85% of patients after NACT and this increase was associated with an improved prognosis [10]. Furthermore, a specific increase in CD8+ T cells and a decrease in CD4+ T cells and B cells [11,12] in post-NACT residual breast tumors is associated with improved survival [13]. HLA-DR is a marker of T cell activation and was recently shown to be increased on CD8+ T cells of different cancer types [14,15]. These cells, which are mainly activated by antigens or cytokines, induce apoptosis in target cells via two main pathways: lytic (cytotoxicity) and non-lytic (cytokine production) mechanisms [16]. Lytic process is based on the secretion of granules containing effector molecules such as granzyme B and perforin, which are released to kill target cells. Non-lytic mechanisms include IFN-γ, TNF-α, and IL-2 secretion, which are crucial for antiviral and inflammatory responses [17]. It has been reported that CD8+ HLA-DR+ T cells in infectious diseases have the ability to produce cytokines such as IFN-γ, TNF-α, and IL-2, and to enhance their cytotoxic activity following stimulation [18,19]. An association has been reported between the plasma levels of IFN-γ and the presence of HLA-DR on CD3+ lymphocytes [20], but the functional ability of these CD8+ HLA-DR+ T cells in cancer patients is still unknown.

It has recently been shown that HLA-DR expression on CD8+ T cells reflects tumor immune status and is associated with a reduction in tumor size and tumor cell migration [14]. Saraiva et al. found that CD8+ HLA-DR+ T cells and Tregs, which are located in intraepithelial tumor structures, were associated with response to NACT. Furthermore, infiltrating CD8+ HLA-DR+ T cells were shown to play an essential role in anti-tumor response and to be elevated in patients without axillary lymph node metastasis who have a good capacity for response to NACT [7].

To explore the role of CD8+ HLA-DR+ T cells in HER2 negative BC patients after NACT, we firstly compared the percentage of these cells in patients who responded and who did not respond to NACT. Our goal was to decipher the effector molecules of this subpopulation and soluble factors that can activate it. To achieve this objective, we firstly analyzed the influence of plasma content on the induction of effector cytotoxic functions, activating healthy donor CD8+ HLA-DR+ T cells in the presence of plasma from responder (R) and non-responder (NR) patients. Then, after comparing the plasmatic concentration of IFN-γ and IL-12 in R and NR patients’ plasma, we neutralized the effect of these differentially expressed cytokines in activating cultures.

## 2. Materials and Method

### 2.1. Patients and Samples

Peripheral blood from 18 healthy donors (HD), as controls, and 73 BC patients (post-NACT) were collected in Vacutainer tubes with heparin (BD Biosciences, San Jose, CA, USA). All patients recruited were HER2 negative and received NACT with antracyclines and taxanes. Patients were selected according to convenience sampling and to the criteria specified below: female patients, 18 years old, stages II-III who were potentially resectable in addition to having an evaluation of pathological response according to the criteria of residual cancer burden (RCB). The expressions of estrogen (ER) and progesterone (PR) receptor were evaluated and patients were classified as ER+ PR+ (DP), ER- PR- (DN) and ER+ PR- or ER- PR+ (SP). A summary of patient characteristics is given in Table 1. Written informed consent was obtained for each patient and ethical approval for the study was granted by the Hospital de la Santa Creu i Sant Pau Institutional Ethics Committee.

Tumor response was evaluated in surgical specimen using the RCB as previously reported [21,22]. Patients who responded to NACT were classified as follows: R for those subjects who achieved a complete pathological response in breast and axilla, (pCR, *n* = 10), those that who had a minimal residual burden (RCB-I, *n* = 6); and NR for patients who maintained a moderate (RCB-II, *n* = 40) or marked (RCB-III, *n* = 17) residual disease post-treatment.

### 2.2. Surface Staining and Analysis by Flow Cytometry

PBMCs were isolated from the heparinized peripheral blood of HD and BC patients using a Ficoll-Histopaque gradient (Lymphoprep, AXIS-SHIELD PoCAs, Oslo, Norway) and were cryopreserved. After thawing, PBMCs were stained with the following anti-human antibodies: CD3-PE (Immunotools, Friesoythe, Germany), CD8-Vioblue (Miltenyi Biotech, Bergisch Gladbach, Germany) and HLA-DR-APC-Cy7 (Miltenyi Biotech). Data acquisition and analysis was performed on a MACSQuant Analyzer 10 flow cytometer (Miltenyi Biotech). For data analysis, live single cells were analyzed to select lymphocytes based on their morphology by forward- versus side-scatter (FSC-SSC) dotplot. By combining anti-CD3 and anti-CD8, we identified CD3+ CD8+ (CD8+ T cells).and CD3+ CD8- (based on our previous results, we were able to infer than 99.5% of CD3+ CD8- cells were CD4+ T cells) [23]. Then, the HLA-DR expression on these T subsets was analyzed resulting in: CD3+ CD8- HLA-DR+, CD3+ CD8- HLA-DR- for CD4+ T cells and CD3+ CD8+ HLA-DR+ or CD3+ CD8+ HLA-DR- for CD8+ T cells. The percentage of HLA-DR positive cells and the mean fluorescence intensity (MFI) of HLA-DR in each subset were obtained using FlowJo version 10 (FlowJo, Ashland, OR, USA).

### 2.3. Cell Culture and Intracellular Phenotyping by Flow Cytometry

Fresh isolated PBMCs from HD were cultured in 96 well-plates (Thermo Fisher Scientific, Vienna, Austria) with 25% of plasma from R and NR patients in RMPI-1640 (Biowest, Nuaille, France) supplemented with 10% FBS and 1% Penicillin/Streptomycin (Biowest) during 72 h in 5% CO2 at 37 °C as previously described [24]. For blocking studies, plasma was pre-incubated 30 for minutes with 10 µg/mL of neutralizing purified mouse anti-human IL-12 mAb (clone C8.6; BD Biosciences) or 0.5 µg/mL of mouse anti-human IFN-γ mAb (clone H21; Genzyme, Boston, MA, USA) as previously reported [25,26]. Four hours before the culture end, cells were stimulated with 50 ng/mL phorbol myristate acetate (PMA, Sigma Aldrich, S. Louis, MO, USA), 500/mL ionomycin (Sigma Aldrich), 3 μg/mL Brefeldin A (BFA; Sigma Aldrich) and Monensin (BD Biosciences).

The percentage of CD8+ HLA-DR+ T cells producing cytokines was measured by intracellular flow cytometry. After surface staining with anti-CD8-PECy-7 (Biolegend, San Diego, CA, USA) CD3-Viogreen (Miltenyi Biotech) and anti-HLA-DR-FITC (Immunotools) antibodies, PBMCs were intracellularly stained with anti-IL-10-PE (eBioscience, San Diego, CA, USA), anti-IFN-γ-APC (BD Biosciences), anti-TNF-α-PE (Biolegend), anti-perforin-APC (Biolegend) or anti-Granzyme-B-PE (eBioscience) antibodies after fixation and permeabilization according to the manufacturer’s instructions (BD Biosciences). Data acquisition and analysis were performed on a MACSQuant Analyzer 10 flow cytometer (Miltenyi Biotech) using FlowJo version 10 (FlowJo, Ashland, OR, USA).

### 2.4. Determination of Plasmatic Concentration of IL-12 and IFN-γ

Concentrations of IL-12 (Immunotools) and IFN-γ (Mabtech, Stockholm, Sweden) from R and NR patients were determined using specific ELISA kits according to the manufacturers’ instructions and using the specific standard curves of recombinant molecules. The limits of detection were as follows: 15.62 pg/mL for IL-12 and 6.25 pg/mL for IFN-γ.

### 2.5. Statistics

Statistical analyses were performed using GraphPad Prism 7 software. Normal data distribution was assessed by the Kolmogorov-Smirnov test. Variables were presented as mean ± SEM or median (interquartile range-IQR-) according to normal or non-normal distribution respectively. Comparisons between the two groups were tested with the Student’s *t*-test (paired or unpaired) or the Mann-Whitney test according to normal distribution. Comparisons of three or more groups were tested with a one-way analysis of variance (ANOVA) and the Bonferroni post-hoc test. Correlation analyses were determined with Pearson’s or Spearman’s correlation according to normal distribution. ROC curve for CD8+ HLA-DR+ T cells were performed to assign a threshold to differentiate R from NR patients. The determined area under the curve, sensitivity and specificity were obtained. The cut-off point for CD8+ HLA-DR+ T cells was determined by a ROC curve analysis, considering the expression value that corresponded to the maximum sensitivity and specificity. *p*-values < 0.05 were considered statistically significant.

## 3. Results

### 3.1. HLA-DR Expression on CD8+ T Cells Was Increased in R Patients

**Figure 1:**
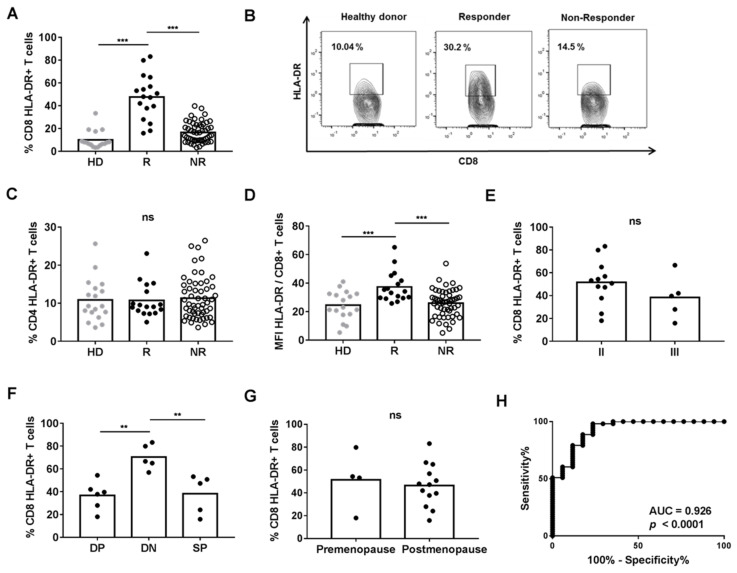
Comparison of HLA-DR expression on T cells from the peripheral blood of HD (*n* = 18) and BC patients (*n* = 73). (**A**) Percentage of CD8+ HLA-DR+ T cells. (**B**) Flow cytometry dot-plot representation of HLA-DR expression on CD8+ T cells (**C**) Percentage of CD4+ HLA-DR+ T cells. (**D**) MFI of HLA-DR expression on CD8+ T cells. (**E**) Percentage of CD8+ HLA-DR+ T cells of R patients classified by the cancer stage, (**F**) Percentage of CD8+ HLA-DR+ T cells of R patients classified by the presence of estrogen (ER) and/or progesterone (PR) hormone receptors (SP: ER +/− and PR +/−; DN: ER− and PR−; DP: ER+ and PR+) (**G**) Percentage of CD8+ HLA-DR+ T cells of R patients classified by menopause status. (**H**) ROC curve analysis of HLA-DR levels on CD8+ T cells. ** *p* < 0.01; *** *p* < 0.001.

We compared the percentage of CD8+ T cells expressing HLA-DR in R and NR patients, and HD. Our results showed that R patients had higher levels of CD8+ HLA-DR+ than NR or HD (Figure 1A,B). However, the percentage of CD4+ HLA-DR+ T cells was comparable between the three groups (Figure 1C). We also found that R patients had higher a MFI of HLA-DR on CD8+ T cells when compared with NR or HD (Figure 1D). However, the MFI of HLA-DR on CD4+ T cells was similar in the three groups (data not shown). There was no correlation between the percentages of CD8+ HLA-DR+ T and CD4+ HLA-DR+ T cells nor between the MFI of HLA-DR on CD8+ T cells or the MFI of HLA-DR on CD4+ T cells.

The percentage of CD8+ HLA-DR+ T cells was not statistically different when R patients were grouped according to cancer stage or menopause status, while this percentage was increased in DN patients when they were grouped by expression of hormone receptors (Figure 1E–G).

To find a statistically valid cut-off point of CD8+ HLA-DR+ T cells to differentiate response to NACT, a ROC curve analysis with all patients was performed, leading to >18.05 as the value for NACT responder patients. The area under the ROC curve was 0.926, sensitivity was 60.38% and specificity was 94.12% (Figure 1H).

### 3.2. Plasma from R Patients Increased Cytotoxic and Pro-Inflammatory Activity of CD8+ HLA-DR+ T Cells

**Figure 2:**
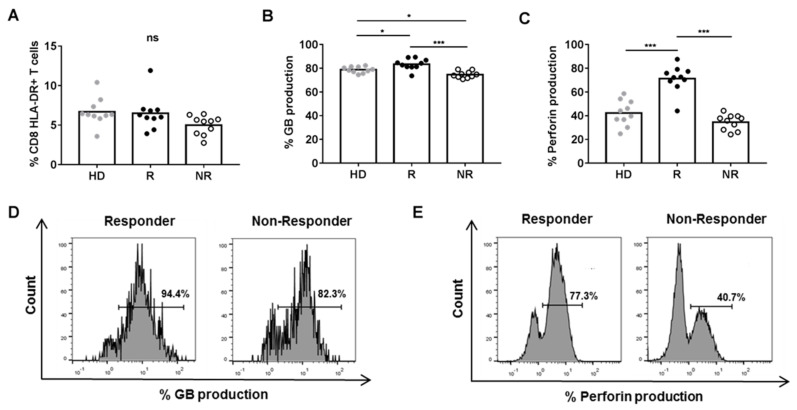
Phenotype and cytotoxic function of CD8+ HLA-DR+ T cells after a 72 h-culture of HD (*n* = 10) PBMCs with 25% plasma from R (*n* = 10) and NR (*n* = 10) BC patients. (**A**) Percentage of CD8+ HLA-DR+ T cells (**B**) Percentage of CD8+ HLA-DR+ T cells producing granzyme B (GB). (**C**) Percentage of CD8+ HLA-DR+ T cells producing perforin. (**D**) Representation of a flow cytometry analysis of granzyme B expression and, (**E**) perforin expression in cultures with plasma from R and NR patients. * *p* < 05; *** *p* < 0.001.

To determine whether plasma soluble factors in BC patients could regulate the percentage and the cytotoxic/pro-inflammatory activity of CD8+ HLA-DR+ T cells, we cultured HD PBMCs in the presence of plasma from HD, R or NR patients. We found that the percentage of CD8+ HLA-DR+ T cells and the MFI of HLA-DR on CD8+ T cells was comparable in the three groups (Figure 2A and data not shown). However, we found a higher percentage of CD8+ HLA-DR+ T cells expressing intracellular granzyme B and perforin when PBMCs were cultured in the presence of plasma from R patients than from HD or NR (Figure 2B–E).

Interestingly, we found a positive correlation between the production of intracellular perforin, but not granzyme B, in CD8+ HLA-DR+ T cells after culturing PBMCs in the presence of plasma from HD, R or NR patients and the percentage of CD8+ HLA-DR+ T cells found in peripheral blood in R and NR patients (Supplementary Figure S1A,B). The production of these cytotoxic molecules was not statistically different between CD8+ HLA-DR− T cells cultured in the presence of plasma from HD, R or NR (Supplementary Figure S2A,B).

**Figure 3:**
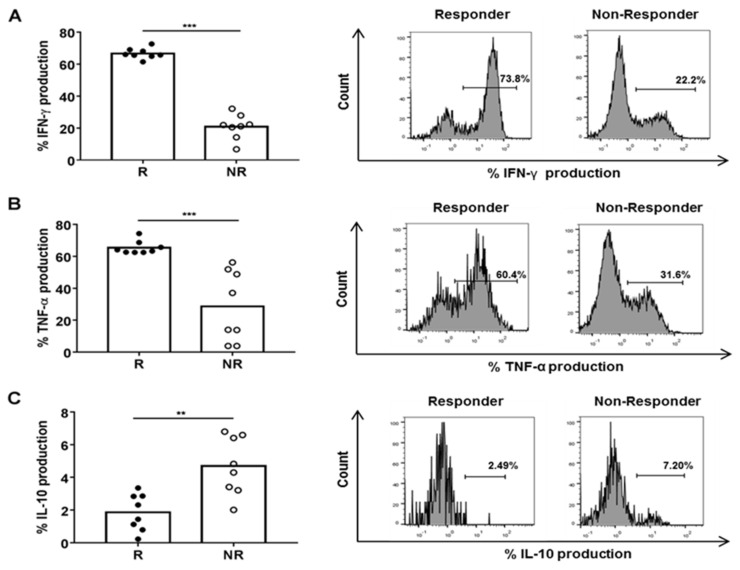
Cytokine production in CD8+ HLA-DR+ T cells after a 72 h-culture of HD PBMCs with 25% plasma from R (*n* = 8) and NR (*n* = 8) BC patients. Percentage of CD8+ HLA-DR+ T cells with intracellular (**A**)IFN-γ, (**B**)TNF-α (**C**) and IL-10 and respective histogram representation. ** *p* < 0.01; *** *p* < 0.001.

The percentage of CD8+ HLA-DR+ T cells with intracellular IFN-γ and TNF-α was higher and IL-10 was lower when PBMCs were cultured in the presence of R than in NR plasma (Figure 3A–C).

We found a positive correlation between the production of IFN-γ, but not of IL-10 and TNF-α, in CD8+ HLA-DR+ T cells after culturing PBMCs in the presence of plasma from R or NR patients and the percentage of CD8+ HLA-DR+ T cells in peripheral blood of all patients (Supplementary Figure S3A–C). However, the production of these cytokines was not statistically different in CD8+ HLA-DR− T cells in the same PBMC culture conditions (Figure S4A–C).

**Figure 4:**
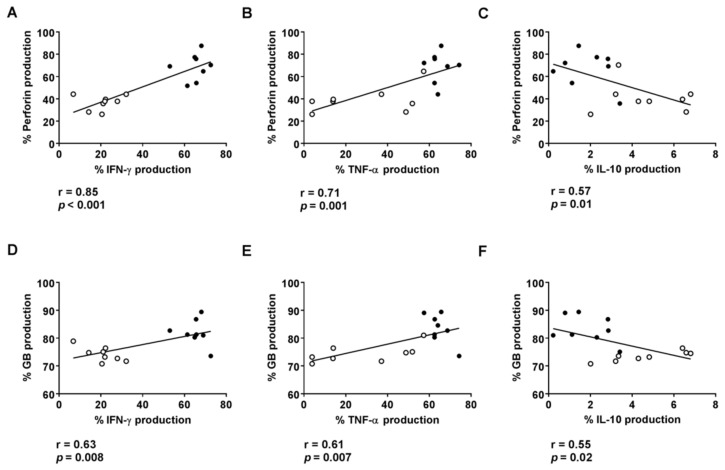
Correlation between the percentages of CD8+ HLA-DR+ T cells with induced intracellular IFN-γ (**A**,**D**), TNF-α (**B**,**E**) or IL-10 (**C**,**F**) and cytotoxic granules after 72 h culture of HD PBMCs with plasma from R and NR patients. Spots in black correspond to R plasma and spots in white to NR plasma.

We also found a positive correlation between the levels of IFN-γ and TNF-α, but a negative correlation between IL-10 levels and the production of perforin and granzyme B by CD8+ HLA-DR+ T cells obtained after culturing PBMCs in the presence of plasma from R or NR patients (Figure 4A–F).

### 3.3. Plasmatic Levels of IL-12 and IFN-γ Are Associated with Cytotoxic Activity on HLA-DR CD8+ T Cells

**Figure 5:**
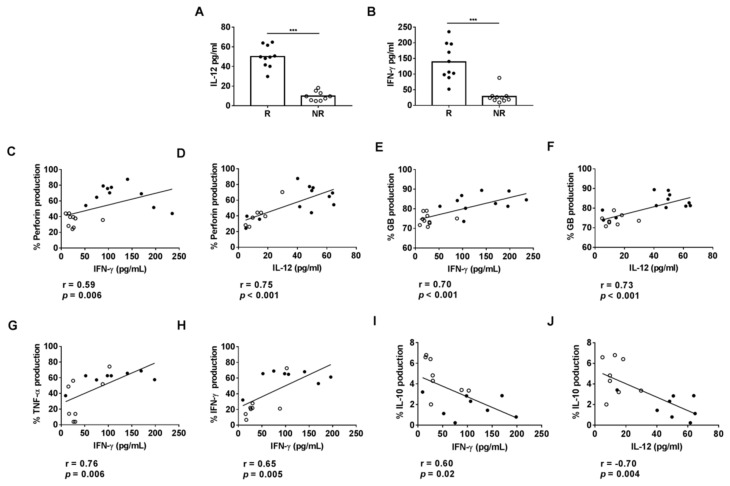
(**A**,**B**) Plasmatic levels of IFN-γ and IL-12 cytokines determined by ELISA in R (*n* = 10) and NR (*n* = 10) patients. (**C**–**J**) Relationship with the percentage of CD8+ HLA-DR+ T cells producing cytokines and cytotoxic granules after 72 h culture of HD PBMCs with plasma from R and NR patients. Spots in black correspond to R plasma and spots in white to NR plasma. *** *p* < 0.001.

To identify plasmatic factors that may contribute to the effector profile of CD8+ HLA-DR+ T cells in the R patients, we compared the plasmatic IL-12 and IFN-γ concentrations of R and NR patients by ELISA. IL-12 and IFN-γ concentrations were significantly higher in R than in NR patients (Figure 5A,B). We also found a positive correlation between plasmatic IL-12 and IFN-γ concentrations with the intracellular percentages of perforin, granzyme B, TNF-α and IFN-γ on CD8+ HLA-DR+ T cells (Figure 5C–H) but a negative correlation with the percentage of CD8+ HLA-DR+ T cells with intracellular IL-10 (Figure 5I,J).

**Figure 6:**
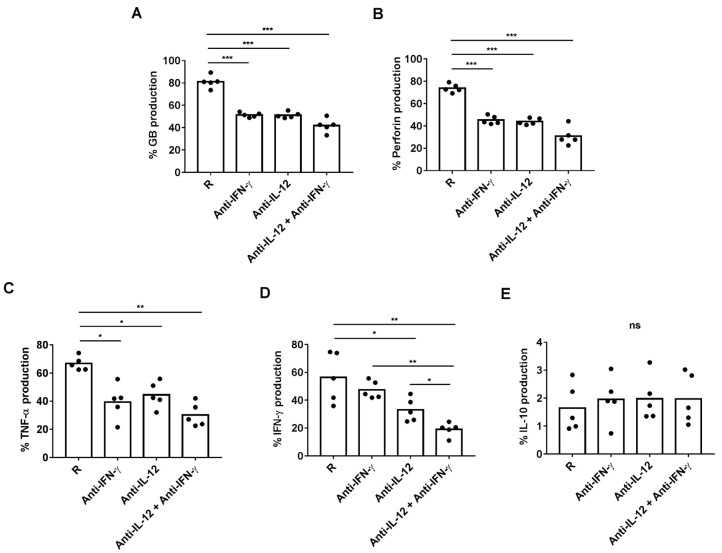
Regulation of cytotoxic granules and intracellular production of cytokines by neutralizing 0.5 ug/mL of anti-IFN-γ (*n* = 5) and/or 10 ug/mL of anti-IL-12 (*n* = 5) antibodies in a 72 h-culture of HD PBMCs with 25% plasma from R patients. Percentages of CD8+ HLA-DR+ T cells producing intracellular (**A**) granzyme B, (**B**) perforin, (**C**) IFN-γ (**D**) TNF-α (**E**) and IL-10 determined by ELISA. * *p* < 0.05; *** *p* < 0.001.

We next investigated whether plasmatic IL-12 and IFN-γ were responsible for inducing the production of effector cytotoxic and pro-inflammatory molecules by CD8+ HLA-DR+ T cells. For this purpose, we added neutralizing anti-IL-12 or anti-IFN-γ antibodies before culturing PBMCs with plasma from R patients. Anti-IL-12 or anti-IFN-γ antibodies, alone and in combination, decreased similarly the percentage of CD8+ HLA-DR+ T cells producing perforin, granzyme B and TNF-α (Figure 6A–C). Interestingly, the combination of anti-IFN-γ with anti-IL-12 antibodies had an additive effect on reducing the production of IFN-γ by CD8+ HLA-DR+ T cells (Figure 6D). None of the neutralizing antibodies had any effect on the IL-10 production by CD8+ HLA-DR+ T cells (Figure 6E).

## 4. Discussion

We observed that CD8+ HLA-DR+ T cells were increased in BC patients who responded to NACT. Plasma from R but not from NR patients can enhance the percentage of intracellular cytotoxic granules and pro-inflammatory cytokines only in CD8+ HLA-DR+ T cells. We found that plasmatic levels of IFN-γ and IL-12 were higher in R than in NR patients and were correlated with the cytotoxic and pro-inflammatory activity of plasma-cultured CD8+ HLA-DR+ T cells. When we blocked plasmatic IFN-γ and IL-12, a significantly decreased percentage of intracellular cytotoxic granules and pro-inflammatory cytokines were found on CD8+ HLA-DR+ T cells. These results suggest that NACT induces an anti-tumor response, activating the effector cytotoxicity of CD8+ T cells through IFN-γ and IL-12.

We observed higher percentages of CD8+ HLA-DR+ T cells in peripheral blood from R patients, suggesting, in line with studies in other neoplasias, an increased activation of these cells [14,15]. We can speculate that this increased CD8+ T cell activation is the consequence of high proliferative tumors where NACT is more effective. Our findings are in concordance with Saraiva et al., who reported that CD8+ HLA-DR+ T cells were increased in BC patients who responded to NACT [7]. In addition, we observed that patients with the highest percentages of CD8+ HLA-DR+ T cells were ER−/PR−. These results suggest the influence of hormone receptors on CD8+ T cell activation. A possible explanation for this is that tumor cells expressing ER and/or PR may regulate CD8+ T cell activation, and consequently, in the absence of hormone receptors, there was a higher activation of CD8+ HLA-DR+ T cells. In line with this, one study reported a mechanism by which ER+ breast cancer tumor cells from breast cancer treated with estrogen upregulated the granzyme B inhibitor proteinase inhibitor-9 (PI-9), which suppresses CD8+ T cell cytotoxic activity [27].

Culturing HD PBMCs with plasma from patients, we found that plasma from R patients did not increase the expression of HLA-DR on CD8+ T cells. However, these experimental conditions cannot rule out the possibility that the upregulation of HLA-DR on peripheral CD8+ T cells may have been occurred in the tumor microenvironment or under tumor influence in R patients. It is tempting to speculate that the increase in HLA-DR expression on CD8+ T cells reflects their activation status and may be induced by released tumor peptides during an effective response to NACT. It is also possible that a longer culture is required to increase the expression of HLA-DR under in vitro conditions. For instance, a higher percentage of activated CD8+ HLA-DR+ T cells after 5 days of culture with HIV peptides has been reported [28]. However, our findings differ from those of Lebossé et al., who showed that following 72 h of culture in the presence of 25% of plasma derived from cirrhotic patients, HLA-DR expression was upregulated on isolated CD8+ T cells from HD. However, the content of plasmatic soluble factors in cirrhotic and BC patients are likely different [24], hence the consequences observed. With our current procedure we cannot rule out that the origin of peripheral CD8+ HLA-DR+ T cells is the tumor microenvironment and, therefore, BC plasma in vitro cannot upregulate HLA-DR expression. In fact, an increased cytotoxic CD8+ T cells in residual breast tumors post-NACT in TNBC patients has been reported [13].

Our results show that plasma from R patients increased the intracellular content of cytotoxic granules and pro-inflammatory cytokines in CD8+ HLA-DR+ T. A possible explanation for this is that the action of NACT generates a pro-inflammatory context that activates an effective cytotoxic response in CD8+ HLA-DR+ T cells from R patients. To confirm the cytotoxic capacity of CD8+ HLA-DR+ T cells after activation with R plasma from BC patients, a co-culture with autologous tumor cells would be necessary. In line with this, a previous report reported CD8+ HLA-DR+ T cell activation with the production of IFN-γ and granzyme B after co-culturing with glioma cells [14]. Moreover, Tassi et al. observed CD8+ HLA-DR+ T cell activation with the production of IFN-γ and IL-2 after co-culturing with neoplastic tumor cells [29].

Here, we have showed that culturing PBMCs with NR plasma induces a higher percentage of CD8+ HLA-DR+ T cells producing intracellular IL-10. It is possible that CD8+ HLA-DR+ T cells from NR patients, without induced cytotoxic activity but with immunosuppressive potential, may be related with tumor progression. These results are in concordance with those of Noble et al., who showed that CD8+ T-cells can secrete anti-inflammatory cytokines, such as IL-10, with suppressor activity [30].

In this study, we have found an increased activity of CD8+ HLA-DR+ T cells in R patients classified as DN. We have also found that plasma IFN-γ and IL-12 levels were comparable between patients were segregated according to the expression of hormone receptors (data not shown). Moreover, no differences in the percentage of CD8+ HLA-DR+ T cells that produce granzyme B or perforin were found (data not shown). We are aware that more samples would be needed to find a definitive conclusion. In addition to this cytotoxic potential, CD8+ HLA-DR+ T cells have been found to have the machinery needed for antigen processing and loading on HLA-DR molecules and, therefore, may express co-stimulatory molecules for an effective T cell activation [31].

Three of our results suggest that plasmatic IFN-γ and IL-12 are partly responsible for induced cytotoxic and pro-inflammatory activity on CD8+ HLA-DR+ T cells. First of all, we determined that plasmatic levels of IFN-γ and IL-12 were higher in R than in NR patients. Second, we found a positive correlation between the percentage of intracellular cytotoxic granules and pro-inflammatory cytokines with the plasmatic levels of IFN-γ and IL-12. Finally, the blocking of these cytokines reduced the percentage of intracellular cytotoxic and pro-inflammatory content on cultured CD8+ HLA-DR+ T cells. It’s difficult to know if circulating plasma levels of IFN-γ and IL-12 are the cause or the consequence of the CD8+ HLA-DR+ T cell activation. It is tempting to speculate that the released tumor peptides during an effective response to NACT can stimulate APCs, which produce IL-12 and activate IFN-γ production on CD8+ HLA-DR+ T cells, as previously reported on CD8+ T cells [32]. Comparison between pre- and post-NACT patients and further studies were needed to understand this mechanism. Accumulating evidence has indicated that IFN-γ and IL-12 are essential for maximal effector CD8+ T cell accumulation and for sustaining effector differentiation [33,34]. However, the functions of IL-12 and IFN-γ were not identical in our neutralizing experiments. In addition, we found that anti-IL-12 or the combination of anti-IL-12 with anti-IFN-γ antibodies, but not anti-IFN-γ antibodies alone, decreased the percentage of intracellular IFN-γ by CD8+ HLA-DR+ T cells. Our findings differ from those of Curtsinger et al., who showed that autocrine IFN-γ signals increase effector IFN-γ production on CD8+ T cells. However, concordantly with our results, several authors have shown that, in certain contexts, IL-12 is sufficient to promote antigen-independent IFN-γ production by CD8+ T cells [35,36,37]. It has been reported that IL-12 induced CD8+ T cells not only express higher levels of cytotoxic granules but also facilitate T cell lysis activity [38]. Since IFN-γ and IL-12 are produced by different cells and they stimulate different cell populations, it is tempting to speculate that they could have an indirect additive or synergistic effect, as previously reported, on CD8+ T cells [32,39,40].

Our findings suggest that plasma levels of IFN-γ and IL-12 could be used as a biomarker to identify responder patients after NACT. In this line, increased levels of plasma IFN-γ only in NACT responder patients on BC patients have already been reported [7]. Additionally, other studies have shown that the presence of IL-12 is a prognostic assay to neoadjuvant chemotherapy in cervical cancer [41]. However, further studies will be needed with more samples, detailed therapies and different cohorts to validate this result.

Despite the contribution of these results to our understanding of the role of this subset of cytotoxic cells, our study has some limitations. One is that samples were obtained at the end of NACT patients were included and the percentage of CD8+ HLA-DR+ T cells in pre-NACT patients is not known. Another is that our findings cannot be extrapolated to primary tumors since the phenotype and the functional mechanisms of CD8+ HLA-DR+ T cells can differ in the tumor microenvironment. Additionally, with our current approach, we cannot predict the function of CD8+ HLA-DR+ T cells with other BC therapies. Finally, the limited number of samples does not allow to analyze subgroups of BC patients.

## 5. Conclusions

Our findings not only contribute to the characterization of the mechanisms involved in the anti-tumor response of CD8+ HLA-DR+ T cells but also suggest that activating and/or expanding these cells could be used for future therapeutic purposes. Particularly, we speculate that increasing IFN-γ and IL-12 could improve response to NACT. We are currently validating the use of the levels of CD8+ HLA-DR+ T cells in another cohort of BC and other tumor patients as a biomarker of response to NACT. Furthermore, it would be interesting to compare the frequencies of CD8+ HLA-DR+ T cells in blood and the primary tumor tissue collected at the same time point.

## Figures and Tables

**Table 1:** Patient demographic and primary invasive tumor characteristics.

Patient Characteristics	Classification	Value
Median age (range), years		58 (36–84)
Menopause status, *n* (%)	Pre-menopause	21 (28.7%)
	Post-menopause	52 (71.2%)
Hormone receptor, *n* (%)	ER	
	+	56 (76.7%)
	−	17 (23.2%)
	PR	
	−	47 (64.3%)
	+	26 (35.6%)
Cancer stage, *n* (%)	II	42 (57.5%)
	III	31 (35.6%)
Ki67, median (range), %		25% (2–85%)
Dimension, median (range), mm		45 (10–120)
Axillary lymph node invasion status, %	Negative	47.3%
	Positive	52.7%
Residual cancer burden (RCB), %	0-pCR	14.9%
	I	8.1%
	II	52.7%
	III	23%

## Data Availability

The data that support the findings of this study are available from the corresponding author upon reasonable request.

## Supplementary Materials

The following are available online at https://www.mdpi.com/article/10.3390/cancers13246167/s1, Figure S1: Correlation between CD8+ HLA-DR+ T cells and the intracellular percentage of perforin (**A**) and granzyme B (**B**), Figure S2: Perforin (**A**) and granzyme B **(B**) production by CD8+ HLA-DR− T cells after culture, Figure S3: Correlation between CD8+ HLA-DR+ T cells and the intracellular percentage of IFN-ϒ (**A**), TNF-α (**B**) and IL-10 (**C**), Figure S4: Intracellular IFN-ϒ (**A**), TNF-α (**B**) and IL-10 (**C)** production in CD8+ HLA-DR− T cells after culture.
