# Supplementary material for: Effector Mechanisms of CD8+ HLA-DR+ T Cells in Breast Cancer Patients Who Respond to Neoadjuvant Chemotherapy
Source: Cancers (Basel). 2021 Dec 7;13(24):6167. doi: 10.3390/cancers13246167 (PMC8699550; doi:10.3390/cancers13246167)
Supplement: Supplementary file 1 [file cancers-13-06167-s001.zip › cancers-1469573-supplementary.pdf]

Supplementary materials

Effector Mechanisms of CD8+ HLA-DR+ T Cells in Breast Cancer Patients Who Respond to Neoadjuvant Chemotherapy

Rubén Osuna-Gómez, Cristina Arqueros, Carla Galano, Maria Mulet, Carlos Zamora, Agustí Barnadas and Silvia Vidal

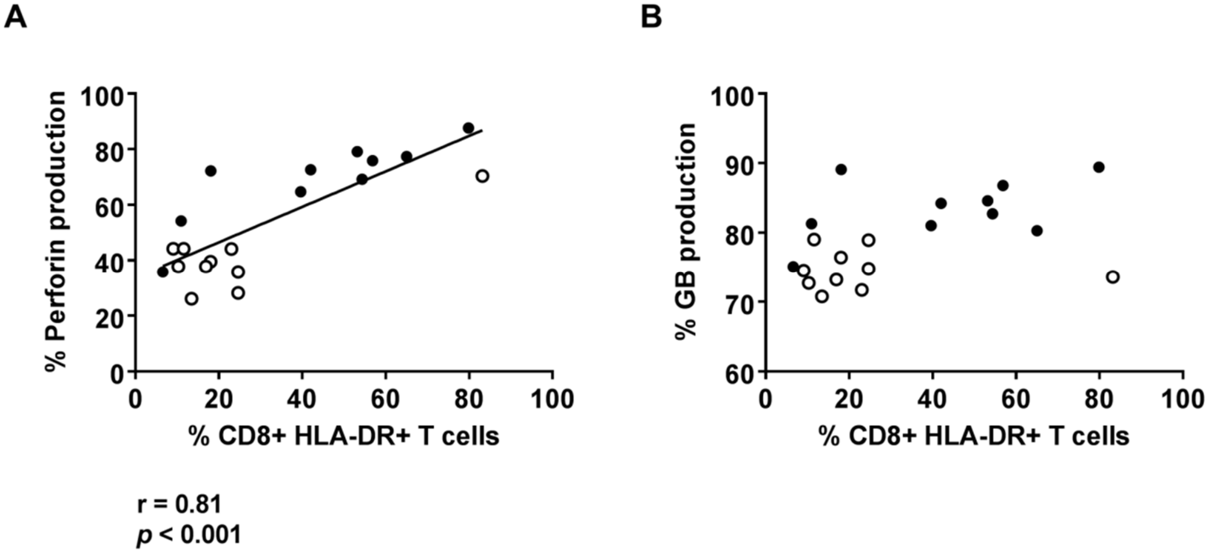

Figure S1. Correlation between CD8+ HLA-DR+ T cells in peripheral blood and the intracellular percentage of (**A**) perforin and (**B**) granzyme B production in cultured healthy donor CD8+ HLA-DR+ T cells. Spots in black correspond to R plasma and spots in white to NR plasma.

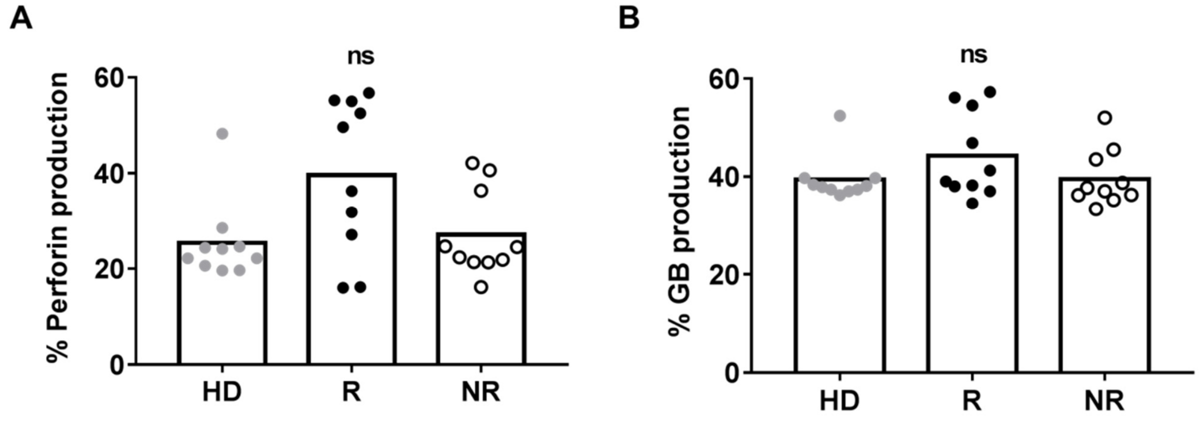

Figure S2. Cytotoxic function of CD8+ HLA-DR- T cells after a 72h-culture of HD PBMCs with 25% plasma from R (n = 10) and NR (n = 10) BC patients. (**A**) Percentage of CD8+ HLA-DR- T cells producing intracellular perforin and (**B**) granzyme B as evaluated by flow cytometry analysis.

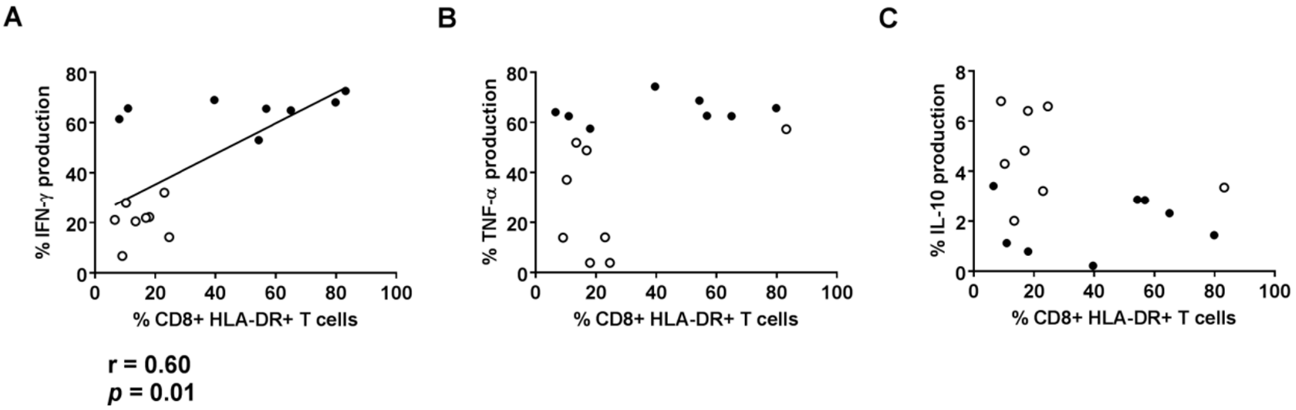

Figure S3. Correlation between CD8+ HLA-DR+ T cells in peripheral blood and the intracellular percentage of (**A**) IFN-γ, (**B**) TNF-α and (**C**) IL-10 production in cultured CD8+ HLA-DR+ T cells. Spots in black correspond to R plasma and spots in white to NR plasma.

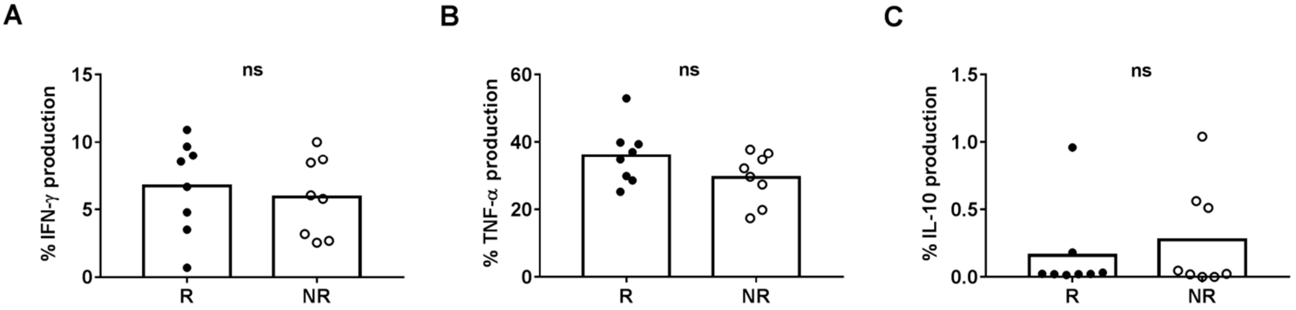

Figure S4. Intracellular cytokine production in CD8+ HLA-DR- T cells after a 72h-culture of HD PBMCs with 25% plasma from R ($n = 8$) and NR ($n = 8$). Percentages of CD8+ HLA-DR- T cells with intracellular (**A**) IFN-γ, (**B**) TNF-α and (**C**) IL-10 were evaluated by flow cytometry analysis.
